# Incised and Punctured Wounds of Joints

**Published:** 1870-05

**Authors:** I. N. Danforth

**Affiliations:** Chicago


Article II.—*Incised and Punctured Wounds of Joints*, by I. N. Danforth, M. D., Chicago.

The surgeon of to-day may well be pardoned if he marvels at the comparatively recent origin, and unaccountably slow growth, of "conservative surgery"—in the largest and best acceptation of the term. Through all the ages, nature has been lavish of her examples of her wondrous power to repair injured parts; to preserve mutilated members; and to give continued and permanent usefulness to organs apparently damaged beyond recovery. And yet, how slow have her disciples been to appreciate this fact, and especially to utilize it, and give it practical application.

We sometimes see most palpable and gratifying instances of this conservative power of nature, in the repair of wounds and injuries of joints; parts which, from the complexity of their mechanism, and the low vitality of some of the tissues which enter into their structure, we should expect would only with great difficulty, and with indifferent success, repair themselves, when severely injured. I have chanced to treat several cases of grave injuries of joints, which have recovered with unexpected rapidity and perfection—three of which I propose to place upon record.

Case i. Daisy C., a bright little girl of eight years, was, with inexcusably culpable carelessness, allowed to amuse herself by running in front of a mowing machine, while the machine was in operation. She suddenly tripped and fell, and her ankle was caught in the machine, and terribly lacerated. I saw the case about an hour afterward. The foot was dangling by the tendo achillis; both malleoli were hanging by shreds of skin, the astragalus was divided so as to deprive it of its upper half, and the tissues generally, in and about the joint, were, to adopt the inelegant but expressive and strictly truthful language of a by-stander, "badly chawed up." The wound might very properly have been called a *contused wound;* and still, the knives of the machine having just been sharpened, there was not very much bruising of the tissues. Bleeding had been arrested by grasping the leg firmly in the hand. A more unpromising case could hardly be imagined; but, the patient presenting every evidence of a sound and vigorous constitution, I determined to give nature a chance to save the foot, and run the risk of being obliged to perform a secondary amputation. All detached and damaged fragments of bone (including both malleoli and half of the astragalus) were carefully removed, many shreds of soft tissue were trimmed away, the arteries ligated, the wound brought together by sutures, the limb placed in a fracture box, and water dressing applied. The only remedies given were an occasional dose of morphia, and one drop of the tincture of aconite every three hours — the latter for several days without intermission. I do not relate the progress of the case in detail, because there is nothing to relate. After the third day, no unfavorable symptoms appeared; the wound healing kindly, and the general symptoms calling for no special care or treatment. Passive motion was commenced very gently and carefully in about three weeks. The result of this case was remarkable. The patient was dismissed after six weeks, with a very slight motion of the joint. A year thereafter I saw her again; she was then walking a mile to school and home again, six days in the week, without the least pain or difficulty, and with scarcely appreciable lameness, and she came bounding toward me with all the agility of childhood. I have recently learned (seven years since the accident) that the foot presents a slightly atrophied appearance, but that locomotion continues all but perfect. The points of interest with regard to this case are, that an injury of a large joint, so extensive and disorganizing, should heal almost entirely by the "first intention," and without suppuration; and that the tissues should so accommodate themselves to the new and novel demands made upon them, as to answer, to a great extent, the purposes of an articulation, in face of the fact that a large proportion of the articular surfaces were entirely and permanently destroyed.

CASE 2. John H., a stout, athletic youth, aged 18, while indulging in the Yankee luxury of "whittling," plunged the blade of a large "jack-knife" into his knee joint to the depth of about one inch and a half. A copious discharge of synovia ensued, the joint became swollen, hot, and painful, and severe febrile symptoms came on. I saw the case about four hours after the accident. At this time the joint was very painful, and there was much constitutional irritation. There was no hemorrhage, but a continuous discharge of synovia from the wound, which was situated on the anterior and external aspect of the right knee. The incision, which was perhaps three-quarters of an inch in length, was carefully closed with strips of muslin dipped in collodion, a liberal coating of collodion was applied over the muslin strips, and the limb kept cool by constant irrigation. The patient was ordered to bed, with strict injunctions to stay there, and, the bowels being confined, a "black dose" was administered — the evacuations to be received in a bed-pan, to obviate the necessity of moving the joint. On the following day, the constitutional symptoms were scarcely lessened, but the wound was much less painful. Opium and aconite were prescribed, with a purgative dose of sulphate of magnesia the next morning. From this time, the case progressed slowly but favorably, until about the eighth day, when scarcely a trace of febrile action remained. I now removed the dressings, and found the wound apparently well united throughout its entire length; but the collodion dressing was renewed, and left on for another week. Opium and aconite were given continuously, in ordinary doses, up to the twelfth day, when I relieved the patient at once of my doses and my attendance. The joint remained swollen, somewhat painful, tender and stiff, for several weeks after the patient's dismissal, but after three months had elapsed, was apparently as sound as ever. I should mention that Tincture of Iodine was applied every second day, for several weeks after the removal of the collodion dressing, and that the articulation was at the same time supported by a bandage.

CASE 3. J. W., a strong laboring man, aged about 35, while "loading" hay, was wounded in the left knee joint by the "tine" of a pitch-fork in the hands of a companion who was "pitching on." The steel prong passed between the extremities of the bones forming the articulation, a little to the left of the patella, to the depth of about two inches. The patient continued his labor for an hour thereafter, although suffering very great pain. I saw the case on the evening of the same day, about six hours after the injury. At that time locomotion was still possible, though productive of great pain; and at every step a peculiar *churning* sound was heard — if carefully listened for — produced by the admixture of the air which had entered the joint, with the synovial fluid, now much increased in consequence of irritation. There was a copious discharge of glairy fluid, (synovia,) from the wound. I looked upon the case as a grave one, in regard to its possible results, and directed my treatment accordingly. A bandage was tightly applied from the junction of the lower with the middle third of the thigh *downwards* to the wound, and another one from the same point on the leg *upwards* to the wound, with the intention of expelling the air from the joint by compression. The wound was now carefully closed with collodion and muslin strips, and cold water constantly applied by irrigation. The patient was sent to bed, an active saline cathartic administered, and the patient forbidden to rise under any circumstances. The further progress of the case presented nothing peculiar. It was simply that of a severe case of traumatic arthritis. For several days the joint was extensively swollen, hot and painful, and active constitutional symptoms were of course present. The collodion dressing was maintained, irrigation was persistently kept up, saline cathartics were administered as occasion required, and the pulse held under control by Fleming's Tincture of Aconite. Recovery was quite slow — presenting in that particular a very marked contrast with that of Case 1 — but in the end very satisfactory. Three months after the date of the injury, the joint was apparently sound, although its mobility was very slightly impeded. In six months, the recovery was quite as complete as is usually obtained after acute rheumatism of the knee joint — and perhaps about as rapid.

In the management and treatment of the foregoing cases, it is not claimed that any new discoveries were made, or any wonderful cures performed. They are related simply as cases somewhat out of the usual class occurring in the practice of an ordinary Practitioner; and as affording excellent illustrations of the power of nature to repair parts complicated in their structure and possessing a low degree of vitality.

